# Optimizing metagenomic next-generation sequencing in CNS infections: a diagnostic model based on CSF parameters

**DOI:** 10.3389/fcimb.2025.1681643

**Published:** 2026-01-20

**Authors:** Xiao-guang Cao, Xiong-feng Zhu, Jun-xi Ni, Hua-dong Meng, Chong-jian Huang

**Affiliations:** 1 Department of Emergency Medical Center, the First Affiliated Hospital of University of Science and Technology of China (Anhui Provincial Hospital), Hefei, Anhui, China; 2 The Third people’s Hospital of Hefei, Hefei, Anhui, China; 3 Department of Emergency Intensive Care Unit (EICU),The Third Affiliated Hospital of Anhui Medical University (The first people’s Hospital of Hefei), Hefei, Anhui, China; 4 Suzhou Hospital of Anhui Medical University (Suzhou Municipal Hospital of Anhui Province), Suzhou, Anhui, China

**Keywords:** CNS infection, CSF, external validation, mNGS, nomogram, predictive model

## Abstract

**Objective:**

This study aimed to assess the association between routine cerebrospinal fluid (CSF) biochemical parameters and metagenomic next-generation sequencing (mNGS) results, and to develop a predictive model to optimize mNGS testing strategies in patients with suspected central nervous system (CNS) infections.

**Methods:**

We retrospectively enrolled 110 patients with suspected CNS infections between December 2019 and January 2024. All underwent both CSF analysis and mNGS testing. Patients were divided into mNGS-positive (n = 62) and negative (n = 48) groups. Logistic regression identified independent predictors, and a nomogram was constructed based on CSF cell count and protein concentration. Model performance was assessed via receiver operating characteristic (ROC) curves, calibration plots, and decision curve analysis (DCA). Internal validation included 10-fold cross-validation and 1000-sample bootstrap. An external validation was performed using a cohort of 40 patients enrolled from another hospital campus (May–October 2024). The derivation cohort was retrospectively collected, whereas the external validation cohort was prospectively enrolled.

**Results:**

mNGS positivity rate was 56.36%, significantly higher than CSF culture (6.36%), with an overall diagnostic concordance of 79.09%. Compared to the mNGS-negative group, positive patients had significantly higher CSF cell counts, protein levels, turbidity, ICU admission (ICUA), antimicrobial regimen adjustment (AAR), and mortality, while glucose was significantly lower (*P* < 0.05). Logistic regression confirmed CSF cell count binary variables (BV) and protein-BV as independent predictors (*P* < 0.05). The areas under curve (AUCs) for the cell-count, protein-only, and combined models were 0.827, 0.813, and 0.782, respectively. Internal validation showed stable results: 10-fold CV AUC = 0.773 ± 0.184 (95% CI: 0.641–0.904), bootstrap AUC = 0.770 ± 0.064 (95% CI: 0.766–0.774). External validation yielded an AUC of 0.763 (95% CI: 0.554–0.918), with sensitivity and specificity of 77.8% and 67.7%. Calibration and DCA demonstrated good agreement and clinical utility.

**Conclusion:**

CSF cell count and protein are reliable predictors of mNGS positivity. The model for practice showed consistent diagnostic performance and may aid in guiding precision mNGS testing, particularly in resource-constrained settings.

## 1 Introduction

CNS infections typically have an acute onset and are associated with high morbidity and mortality. The etiologic spectrum is broad, clinical manifestations are heterogeneous, and microbiological diagnosis remains a significant challenge (Wilson et al., 2019). Current diagnostic approaches primarily rely on CSF culture, polymerase chain reaction (PCR), and neuroimaging, each with notable limitations. CSF culture has a low positivity rate and requires prolonged incubation; PCR, though sensitive, is limited to predefined targets and is ineffective for detecting mixed or rare pathogens. Neuroimaging lacks pathogen specificity (Karagulle-Kendi and Truwit, 2010; Khatib et al., 2017; Vetter et al., 2020). These limitations delay etiological diagnosis, increase misdiagnosis risk, and promote empirical antibiotic overuse and antimicrobial resistance (Li et al., 2020).

The mNGS has emerged as a high-throughput, non-targeted technology capable of detecting a wide range of pathogens—including bacteria, viruses, fungi, and parasites—from a single sample. Compared to traditional methods, mNGS demonstrates higher detection rates and faster turnaround times (within 24 hours) (Miao et al., 2018; Deng et al., 2022; Zhang et al., 2024), and is especially effective for identifying difficult-to-culture or rare organisms such as *Mycobacterium tuberculosis* and neurotropic viruses (Benoit et al., 2024). Despite its advantages, clinical implementation of mNGS remains limited due to high cost, complex bioinformatics workflows, lack of standardized protocols, and potential for false-positive results (Guo and Zhang, 2024; Su et al., 2024; Zhang et al., 2024). Therefore, judicious selection of candidates for mNGS testing is essential to optimize its diagnostic yield and cost-effectiveness.

Previous studies have reported associations between mNGS positivity and routine CSF indicators, such as elevated white blood cell count, increased protein, decreased glucose, and CSF turbidity (Miao et al., 2018; Li et al., 2020). Several groups have attempted to construct predictive models to identify patients most likely to benefit from mNGS testing; however, these models differ in design, included parameters, and validation strategies, which limits their generalisability. We hypothesised that routine CSF parameters could serve as independent predictors of true mNGS positivity and that a simple prediction model based solely on these variables could reliably stratify the pre-test probability of mNGS positivity and guide precision-oriented mNGS testing. Therefore, the primary objective of this retrospective study was to evaluate the association between routine CSF biochemical parameters and mNGS results in patients with suspected CNS infections and to develop and validate a CSF-based predictive model using both internal and external cohorts to support evidence-based decision-making for mNGS testing in clinical practice.

## 2 Materials and methods

### 2.1 Data collection

A total of 135 patients with suspected CNS infections admitted to a tertiary hospital in Anhui Province between December 2019 and January 2024 were initially screened. After applying the inclusion criteria, 110 patients were enrolled in the final derivation cohort for model development. In parallel, an independent external validation cohort consisting of 40 patients was prospectively enrolled between May and October 2024 from another campus of our hospital. Identical inclusion and exclusion criteria, testing methods, diagnostic standards, and analytical procedures were applied to ensure consistency across cohorts. No relevant policy or reimbursement changes occurred during this period. Clinical data for both cohorts were independently collected by two physicians and verified by a senior attending physician. Baseline variables included: age, sex, CSF cell count, albumin, chloride, glucose levels, CSF culture results, whether immediate mNGS testing was performed (immediate mNGS evaluation, INE), whether empirical antimicrobial therapy was administered (EAT), appropriateness of the antimicrobial regimen (AAT), whether AAR, ICUA, length of hospital stay (LHS), Hefei City medical insurance fees (HFMIF), and total hospitalization expenses (HE), among others. All information was extracted from the Donghua Electronic System. For key biomarkers such as CSF cell count, glucose, and albumin, both continuous variables (CV) and BV were analyzed to comprehensively evaluate their diagnostic value in predicting mNGS positivity. The dichotomous thresholds for CSF variables were defined as follows: CSF cell count >10×10^6^/L, protein >0.6 g/L, glucose <2.2 mmol/L, or a CSF-to-blood glucose ratio <0.4. These cutoffs were based on the 2021 Chinese expert consensus on the diagnosis and treatment of central nervous system infections (The Neurological Critical Care Expert Committee of the Neurosurgery Branch of the Chinese Medical Association, and the Neurosurgery Critical Care Group of the Neurosurgery Branch of the Beijing Medical Association, 2021). Considering that reference ranges may vary across different analyzers and reagents, when the reference intervals provided by individual laboratories did not fully coincide with the above values, each variable was dichotomized as “normal” or “abnormal” according to the laboratory-specific reference ranges. Final clinical diagnoses were independently determined by a panel of three senior specialists who were fully blinded to the mNGS results. Diagnoses were based on a predefined composite reference standard that integrated clinical presentation (e.g., fever >38°C, headache, meningeal signs), CSF parameters (e.g., cell count >10 × 10^6^/L, protein >0.6 g/L, glucose <2.2 mmol/L or CSF/serum glucose ratio <0.4 for bacterial meningitis; normal or mildly elevated protein for viral infection), conventional microbiology (culture, Gram stain), targeted PCR assays, neuroimaging (CT/MRI showing enhancement or abscess), response to therapy, and follow-up outcomes, in accordance with the 2021 Chinese Expert Consensus (van de Beek et al., 2016; Tunkel et al., 2017; The Neurological Critical Care Expert Committee of the Neurosurgery Branch of the Chinese Medical Association, and the Neurosurgery Critical Care Group of the Neurosurgery Branch of the Beijing Medical Association, 2021).

### 2.2 Inclusion and exclusion criteria

**Figure 1:**
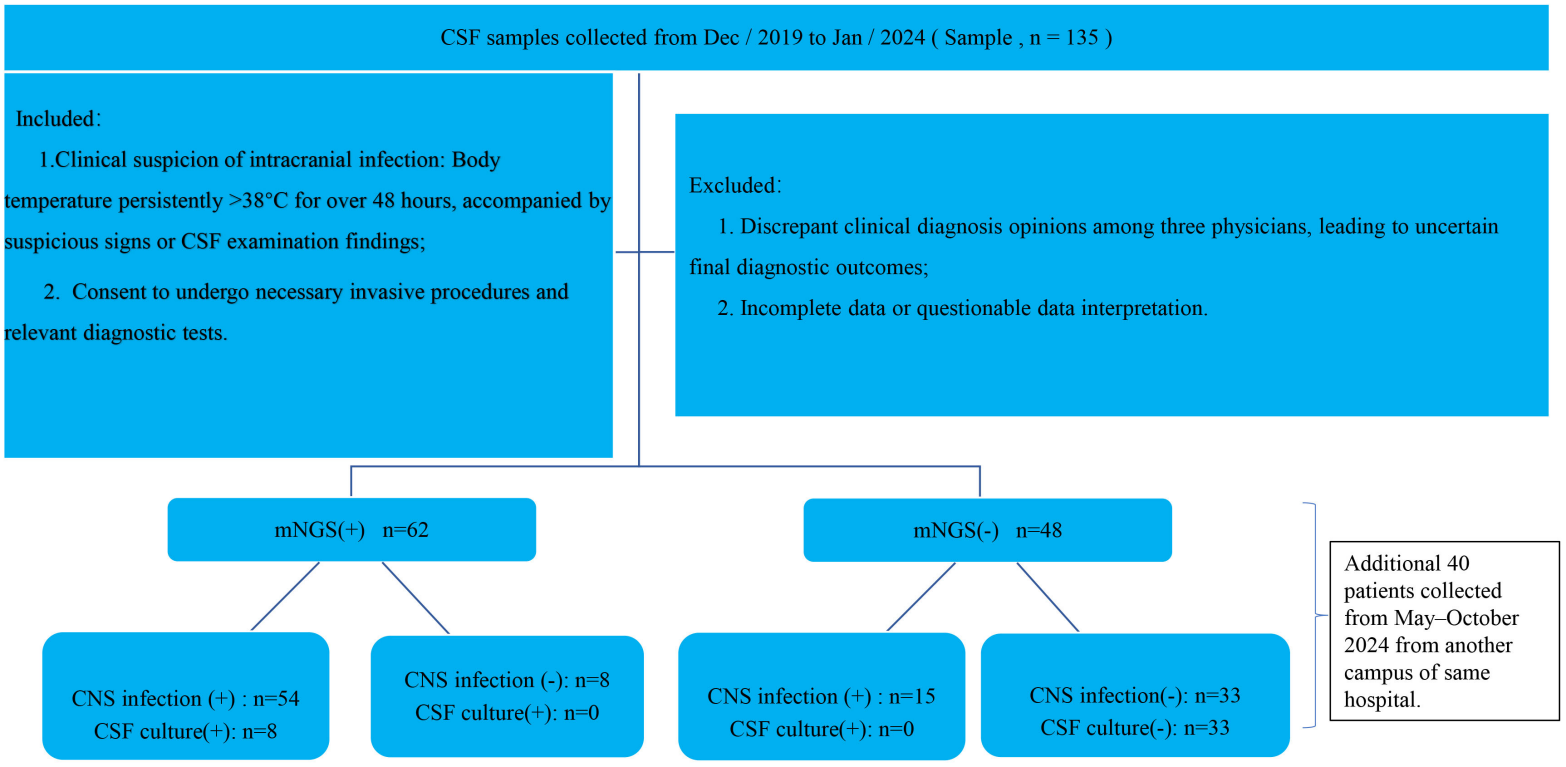
Flow chart of the study.

Inclusion criteria were as follows: (1) clinically suspected intracranial infection defined as a persistent body temperature >38 °C for more than 48 hours accompanied by suspicious signs or abnormal CSF findings; (2) patients or legal guardians provided informed consent for necessary invasive procedures and diagnostic evaluations. Exclusion criteria included: (1) inconsistent diagnostic opinions among the three physicians, resulting in diagnostic uncertainty; (2) incomplete or ambiguous data that hindered clinical interpretation. All enrolled patients and/or their guardians provided written informed consent after being fully informed of the testing protocols (**Figure 1**).

## 3 Clinical diagnosis of CNS infections

Final clinical diagnoses of CNS infections were adjudicated by a panel of three senior specialists (two neurologists and one infectious disease expert) using a pre-specified composite reference standard. The panel independently reviewed all available routine clinical information, including presenting symptoms and signs, CSF cytology and biochemistry, conventional microbiologic tests (CSF and blood cultures, Gram stain, India ink, antigen tests, targeted PCR), blood cultures and serologic assays, neuroimaging (brain CT/MRI), and response to antimicrobial and adjunctive therapy during hospitalisation and follow-up, in accordance with the 2021 Chinese Expert Consensus on CNS infections and international IDSA/ESCMID guidelines (van de Beek et al., 2016; Tunkel et al., 2017; The Neurological Critical Care Expert Committee of the Neurosurgery Branch of the Chinese Medical Association, and the Neurosurgery Critical Care Group of the Neurosurgery Branch of the Beijing Medical Association, 2021). Importantly, all adjudicators were fully blinded to mNGS results throughout this process, and any discrepancies were resolved at a consensus meeting.

CNS infection was defined according to these guidelines as a compatible clinical syndrome plus supportive CSF and/or microbiologic and imaging evidence. In brief, definite or probable bacterial meningitis required typical clinical features with CSF pleocytosis and biochemical changes consistent with bacterial infection, together with microbiologic confirmation from CSF or blood or a characteristic clinical course with response to appropriate antibacterial therapy. Viral meningoencephalitis required compatible clinical manifestations with lymphocytic CSF pleocytosis and/or mild protein elevation, supported by positive viral PCR or serology, typical MRI findings, or a self-limiting course. Non-infectious CNS disorders (e.g. autoimmune encephalitis, cerebrovascular disease, metabolic encephalopathy) were diagnosed according to the same guidelines and specialist consensus in the absence of evidence for CNS infection.

## 4 Study procedures

### 4.1 Conventional testing

All patients underwent lumbar puncture, and 3~5 ml of CSF was collected. Routine analysis included cytological examination, biochemical assays, Gram staining, and bacterial culture. If CSF samples could not be sent for culture within the designated time, 3 ml of CSF was aseptically injected into an aerobic blood culture bottle and submitted to the microbiology laboratory within 2 hours at room temperature for incubation.

### 4.2 mNGS testing and interpretation of results

Following standard procedures, 1.5~3 ml of CSF was collected from each patient via lumbar puncture. A total of 0.6 ml of the CSF sample was mixed with 250 μl of 0.5 mm glass beads in a 1.5 ml microcentrifuge tube, which was mounted on a horizontal vortex mixer and vigorously shaken at 2800~3200 rpm for 30 minutes to achieve mechanical disruption. Subsequently, 7.2 μl of lysozyme was added for enzymatic lysis. After lysis, 0.3 ml of the processed sample was transferred into a new 1.5 ml microcentrifuge tube. DNA was extracted using the TIANamp Micro-DNA Kit (DP316, Tiangen Biotech, China) following the manufacturer’s instructions. For RNA extraction, the TIANamp Micro-RNA Kit (DP431, Tiangen Biotech, Beijing, China) was used. The extracted RNA was reverse transcribed into single-stranded cDNA, and then double-stranded cDNA was synthesized using the PMseq™ RNA High-Throughput Pathogen Detection Kit according to the manufacturer’s protocol. Library preparation included DNA fragmentation, end repair, adapter ligation, and PCR amplification. The quality of the constructed libraries was assessed using the Agilent 2100 Bioanalyzer. Qualified libraries were pooled and converted into DNA nanoballs (DNBs), followed by sequencing on the BGISEQ-50 or MGISEQ-2000 platform. Negative controls were included in each sequencing batch for quality assurance. Interpretation of mNGS results was based on current national guidelines and expert consensus, and clinical correlation was used to determine the significance of detected organisms (Li and Durbin, 2009; O’Leary et al., 2016; Ramachandran and Wilson, 2020).

## 5 Statistical analysis

All statistical analyses and data visualization were performed using R software. Continuous variables were assessed for normality. Normally distributed variables are expressed as mean ± standard deviation (SD), and non-normally distributed variables as median with interquartile range [*M (P25, P75*)]. Categorical variables are summarized as counts and percentages [n (%)].Between-group comparisons were performed using appropriate tests, including the independent samples t-test, Mann–Whitney *U* test, or chi-square (χ²) test. Logistic regression was used to identify independent predictors of mNGS positivity. Model performance was evaluated using ROC curves and the AUC. In addition to the continuous forms of CSF cell count and CSF protein, we also created cutoff-based binary variables for sensitivity analyses. The optimal cut-off for cell count was identified from ROC analysis by maximizing the Youden index.Model calibration and clinical utility were assessed via calibration curves and DCA, respectively. Internal validation was performed using 10-fold cross-validation and bootstrap resampling (1,000 iterations). A two-sided *P* value <0.05 was considered statistically significant. Missing data were excluded from multivariable analysis.

## 6 Results

### 6.1 Baseline characteristics of patients

A total of 135 patients with suspected CNS infections were initially screened, and 110 patients met the inclusion criteria and were enrolled in the final analysis, comprising 73 males and 37 females. Based on mNGS results, patients were divided into a positive group (n = 62) and a negative group (n = 48). The true positive rate and true negative rate of mNGS for diagnosing CNS infections were 78.26% and 80.49%, respectively, with an overall concordance of 79.09% with clinical diagnosis. Compared with the mNGS-negative group, patients in the mNGS-positive group had significantly higher rates of ICUA, mortality, and AAR. In CSF analysis, the positive group also showed a higher proportion of turbidity, elevated cell counts, and increased protein levels, while glucose levels were significantly lower (*P* < 0.05) (**Table 1**).

**Table 1:** Univariate analysis between mNGS-positive and mNGS-negative groups(n=110).

Variables	mNGS(+) (n = 62)	mNGS(-) (n = 48)	*Z/t/χ²*	*P*
Age(year)	53.500 (47.000, 64.750)	53.000 (32.000, 65.000)	1.230	0.219
Gender(M/F)	43 (69.355)/19 (30.645)	30 (62.500)/18 (37.500)	0.570	0.450
INE(Y/N)	34 (54.839)/28 (45.161)	32 (66.667)/16 (33.333)	1.577	0.209
CSF infection(Y/N)	54(87.097)/8(12.903)	15(31.250)/33(68.750)	36.090	<0.001
AAR(Y/N)	36 (58.065)/26 (41.935)	16 (33.333)/32 (66.667)	6.639	0.010
EAT(Y/N)	42 (67.742)/20 (32.258)	36 (75.000)/12 (25.000)	0.691	0.406
Prognosis(S/D)	36 (58.065)/26 (41.935)	38 (79.167)/10 (20.833)	5.472	0.019
ICUA(Y/N)	42 (67.742)/20 (32.258)	23 (47.917)/25 (52.083)	4.399	0.036
HFMIF(Y/N)	17 (27.419)/45 (72.581)	8 (16.667)/40 (83.333)	1.781	0.182
LHS (day)	21.000 (9.250, 35.000)	16.000 (9.000, 25.250)	1.034	0.301
HE (10000.00 yuan)	6.975 (1.811, 17.857)	4.099 (1.609, 11.228)	1.504	0.133
CSF				
Turbidity(Y/N)	36 (58.065)/26 (41.935)	17 (35.417)/31 (64.583)	5.558	0.018
Cell-BV(A/N)	54 (87.097)/8 (12.903)	27 (56.250)/21 (43.750)	13.261	<.001
Cell count-CV(10 × 10^6^/L)	45.500 (38.000, 455.750)	12.500 (5.000, 41.000)	4.684	<.001
Chloride-BV(A/N)	29 (46.774)/33 (53.226)	16 (33.333)/32 (66.667)	2.022	0.155
Chloride-CV()	119.605 ± 7.309	122.025 ± 6.147	1.844	0.068
Glucose-BV(A/N)	51 (82.258)/11 (17.742)	31(64.583)/17 (35.467)	4.454	0.035
Glucose-CV(mmol/L)	2.925 (1.958, 3.987)	3.615 (2.848, 4.505)	2.559	0.011
Protein-BV(A/N)	54 (87.097)/8 (12.903)	30 (62.500)/18 (37.500)	9.068	0.003
Protein-CV(g/L)	1.310 (0.642, 2.353)	0.675 (0.500, 1.225)	3.011	0.003

Y/N indicates Yes or No; S/D, Survived or Deceased; A/N, Abnormal or Normal; +/-, positive or negative, wanyuan:.

### 6.2 Correlation and concordance between mNGS and CSF culture

**Figure 2:**
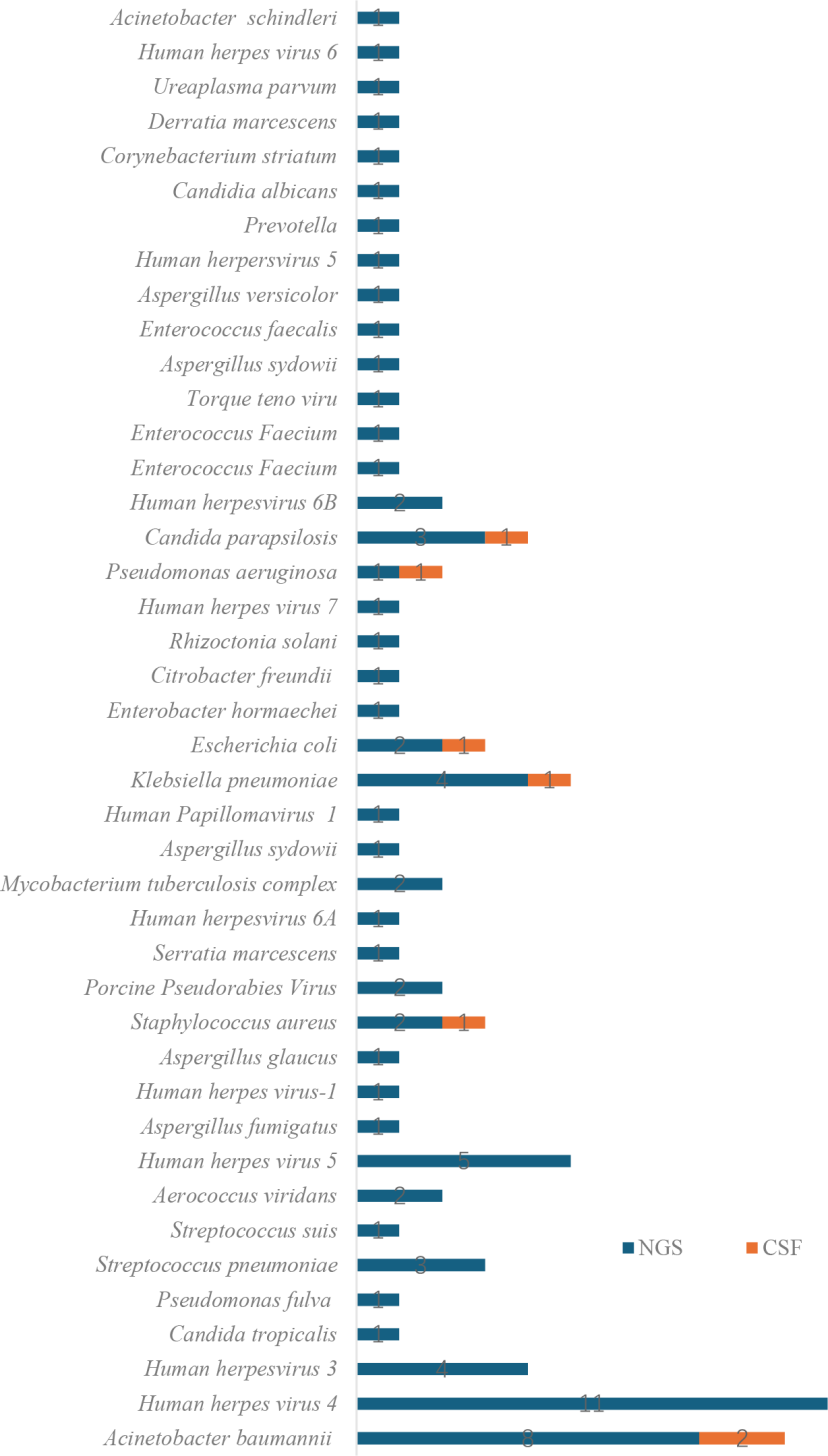
Distribution of pathogens identified by mNGS and CSF culture(n=110).

Among the 110 CSF samples tested by conventional culture, only 7 samples (6.36%) yielded positive results. The identified pathogens included *Klebsiella pneumoniae* (n = 1), *Acinetobacter baumannii* (n = 3), *Escherichia coli* (n = 1), *Staphylococcus aureus* (n = 1), *Pseudomonas aeruginosa* (n = 1), and *Candida tropicalis* (n = 1). All culture-positive samples contained only a single pathogen. In contrast, mNGS identified 80 pathogens in 62 positive cases, with six samples containing two or more pathogens. The most frequently detected pathogens by mNGS were *Acinetobacter baumannii* (n = 8), *Human herpesvirus 5* (n = 11), and *Klebsiella pneumoniae* (n = 4) (**Figure 2**). Moreover, the turnaround time for mNGS was significantly shorter than that of CSF culture. mNGS results were available within 24 hours, while CSF culture required at least 72 to 120 hours.

### 6.3 Logistic regression analysis

**Figure 3:**
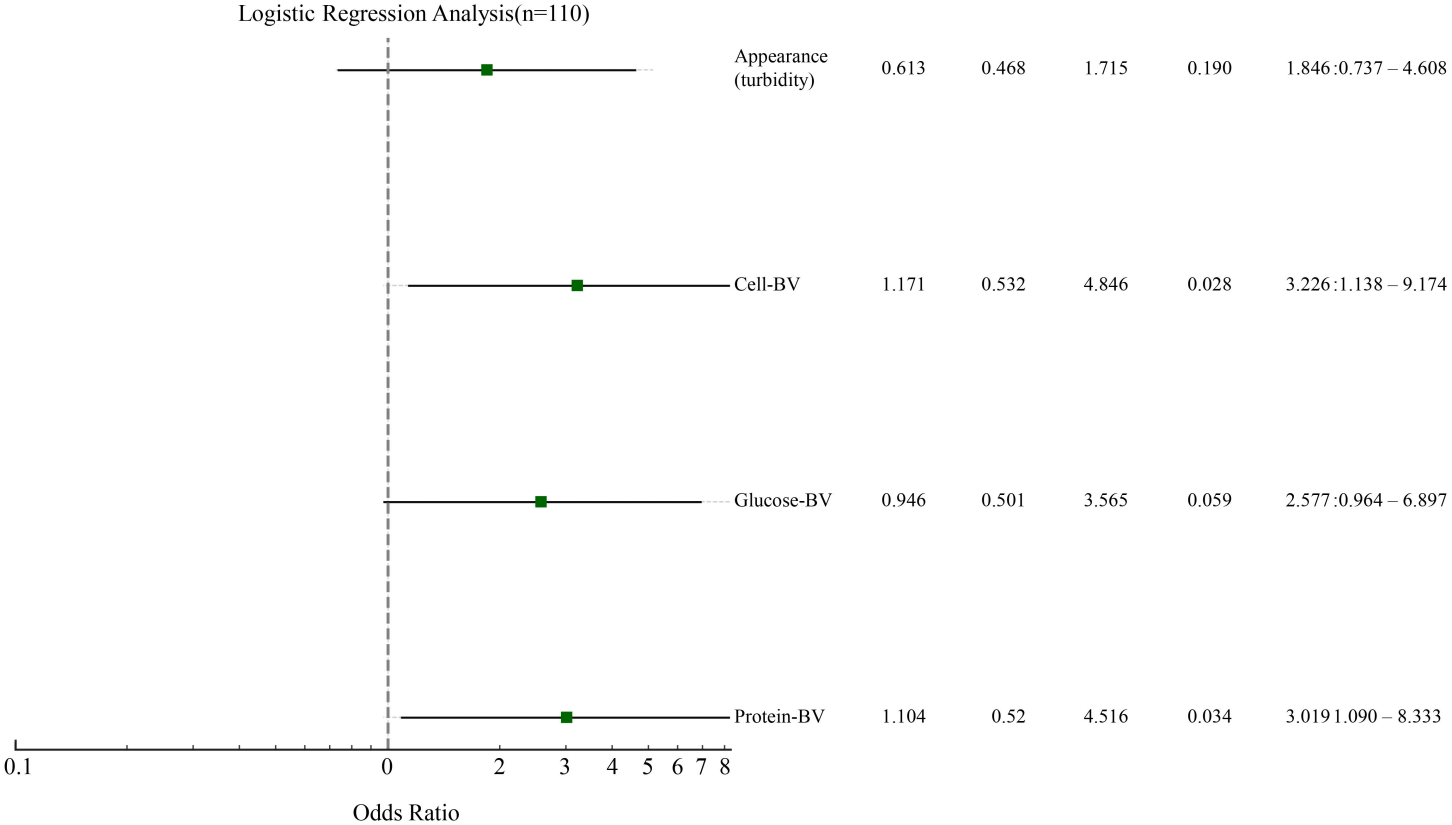
Forest plot of independent predictors associated with mNGS positivity based on multivariable logistic regression analysis.

**Figure 4:**
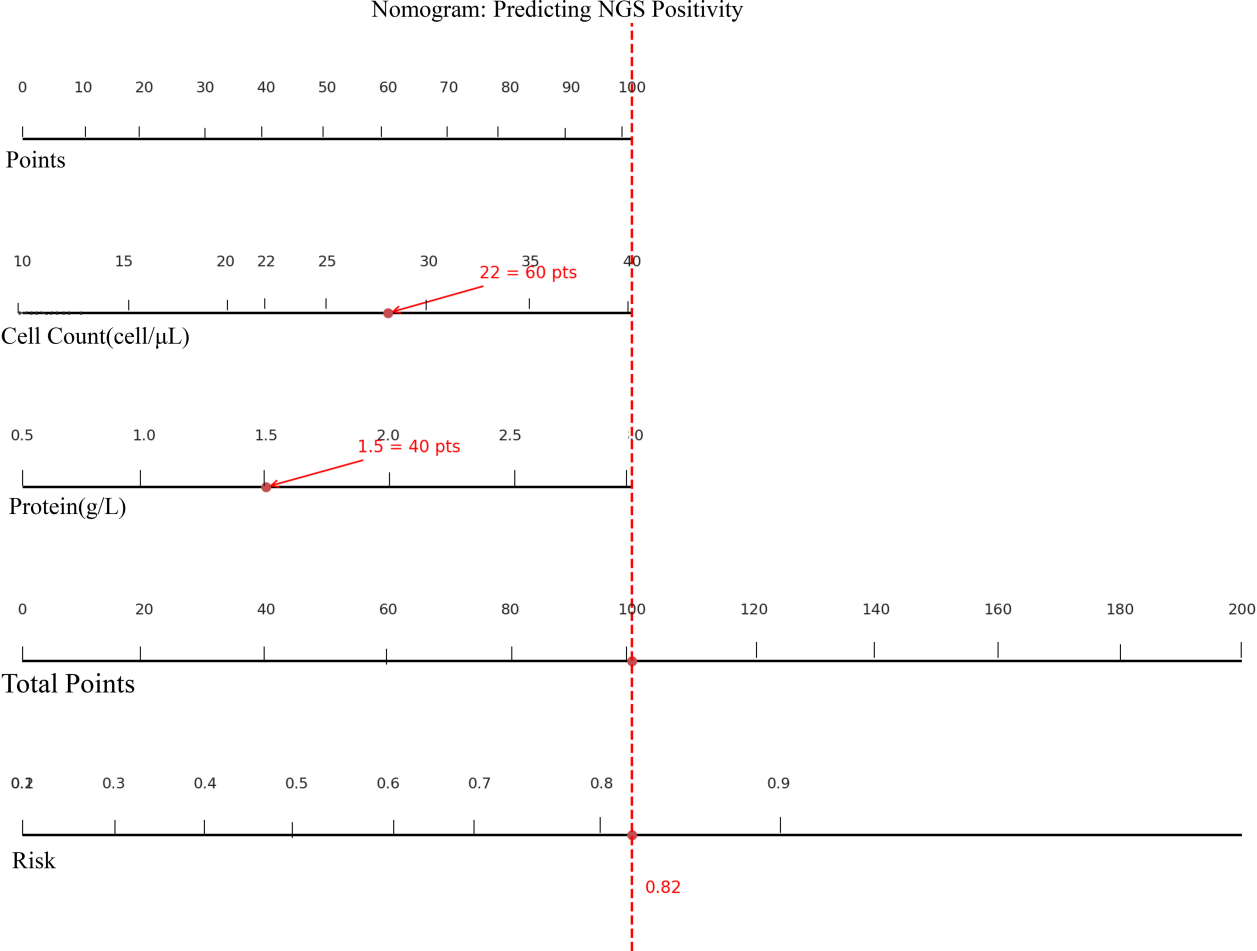
Nomogram for predicting the probability of CSF mNGS positivity.

In clinical practice, CSF laboratory parameters are frequently used as an initial screening tool for suspected central nervous system infections. Accordingly, in the exploratory analysis, CSF cell count, protein concentration, turbidity, and glucose levels were modeled both as CVs and as BVs based on guideline-recommended cutoffs (normal vs abnormal). When specified as BVs, abnormal CSF cell count and elevated CSF protein showed the strongest and most consistent associations with mNGS positivity (**Figure 3**). To assess potential multicollinearity among these BVs, we constructed an intermediate multivariable logistic regression model including all four BVs and calculated variance inflation factors (VIFs). The VIF values were 1.250 for CSF turbidity 1.300 for dichotomized CSF cell count-BV 1.300 for dichotomized CSF glucose-BV, and 1.390 for dichotomized CSF protein -BV, indicating no relevant multicollinearity (all VIF < 2). In addition, auxiliary linear regression models were used to examine collinearity among the corresponding CVs, yielding VIFs of 1.078 for cell count-CV, 1.030 for protein-CV, 1.415 for chloride-CV, and 1.442 for glucose-CV, again suggesting an absence of problematic collinearity.In the intermediate multivariable logistic regression model(enter)that included all four BVs simultaneously, only CSF cell count-BV and CSF protein-BV remained independently associated with mNGS positivity, whereas the other predictors lost statistical significance after mutual adjustment. Based on these findings—and to balance predictive performance, model parsimony, and clinical interpretability—we retained CSF cell count and CSF protein as the core predictors in the final model. These two parameters were therefore selected as key predictors for constructing the final multivariable logistic regression model, in which they were retained in their continuous forms to preserve information and facilitate nomogram development (**Figure 4**). In this final model, both predictors were entered as CVs to preserve information and facilitate nomogram development (**Figure 4**), and with 58 mNGS-positive events and 2 predictors (EPV = 29:1), the risk of overfitting was considered low.

### 6.4 Comparative analysis of diagnostic accuracy among different indicators

**Figure 5:**
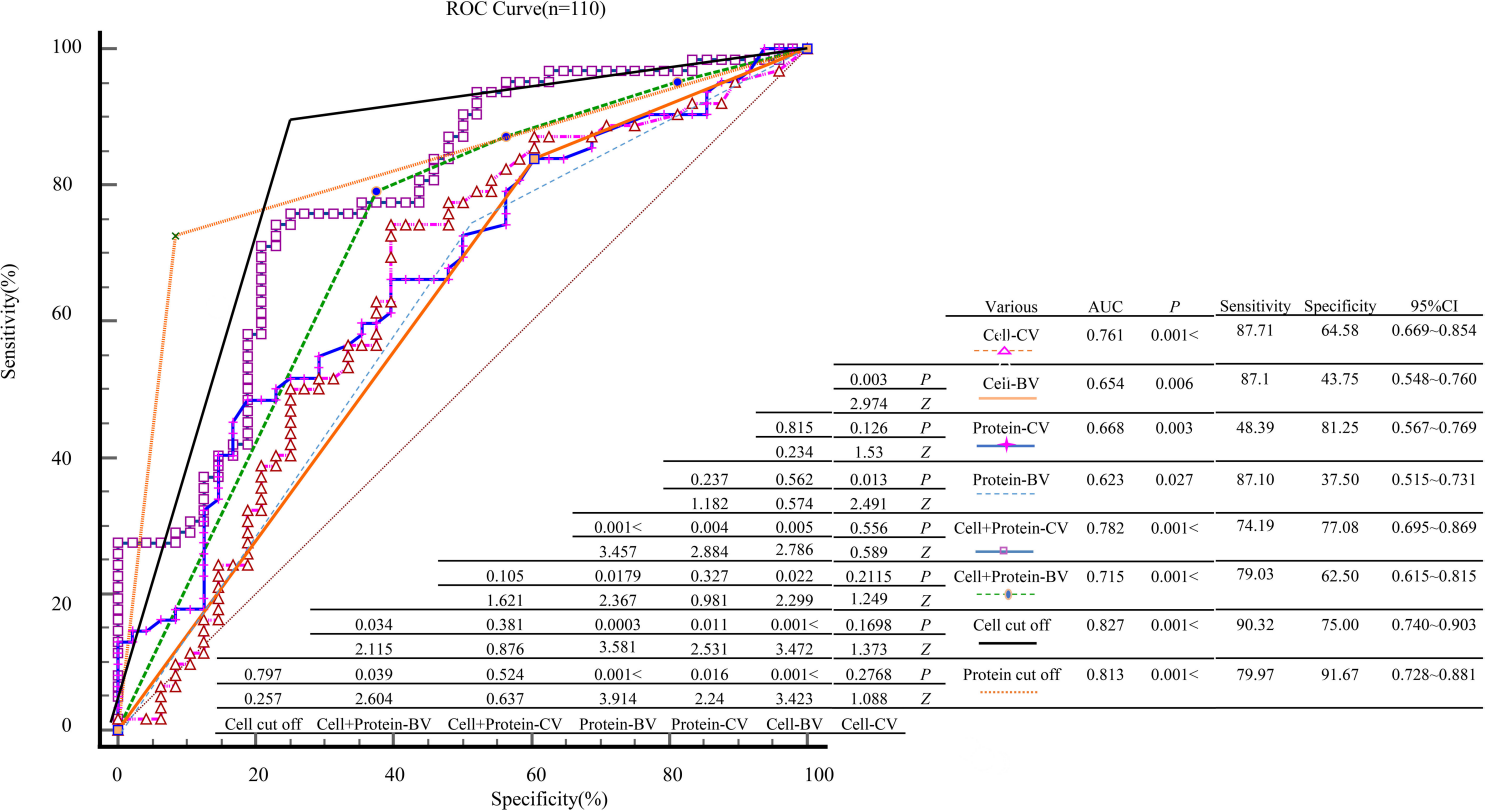
ROC analysis and statistical comparison of diagnostic performance among different indexes. “Cell cut-off” and “Protein cut-off” denote binary variables derived from the optimal ROC-based thresholds, with CSF cell count <21×10^6^/L vs. ≥21×10^6^/L and CSF protein <1.5 g/L vs. ≥1.5 g/L coded as 0 and 1, respectively. Guideline-based BV (Cell-BV, Protein-BV) were used to identify independent predictors in regression analysis, whereas ROC-derived ‘cut-off’ BV (cell cut-off, protein cut-off) were used only for exploratory ROC comparisons.

To evaluate the diagnostic performance of various indicators for predicting mNGS positivity, ROC curves were generated for CSF parameters including cell count, glucose, and protein levels. Each variable was analyzed in both continuous and binary formats, and comparative analyses were performed between different forms of the same variable. The results showed that the cell count cut-off-BV and protein cut-off-BV models had the highest area under the AUC, at 0.827 and 0.813 respectively, indicating excellent sensitivity and specificity. Among the combined models, cell + protein-CV and cell+ protein-BV also demonstrated strong discriminatory ability, with AUCs of 0.782 and 0.715, respectively. Further comparison using DeLong’s Z-test revealed that the cell cut-off, protein cut-off, cell-CV, and cell + protein-CV models had significantly higher diagnostic accuracy than cell-BV, protein-CV, and protein-BV models (*P* < 0.05). Additionally, the combined cell + protein models (both CV and BV formats) showed significantly better performance than the corresponding protein-only models (*P* < 0.05). No statistically significant differences were found among the remaining pairwise comparisons (*P* > 0.05). Detailed results are illustrated in **Figure 5**.

### 6.5 Internal and external validation

#### 6.5.1 Internal validation

**Figure 6:**
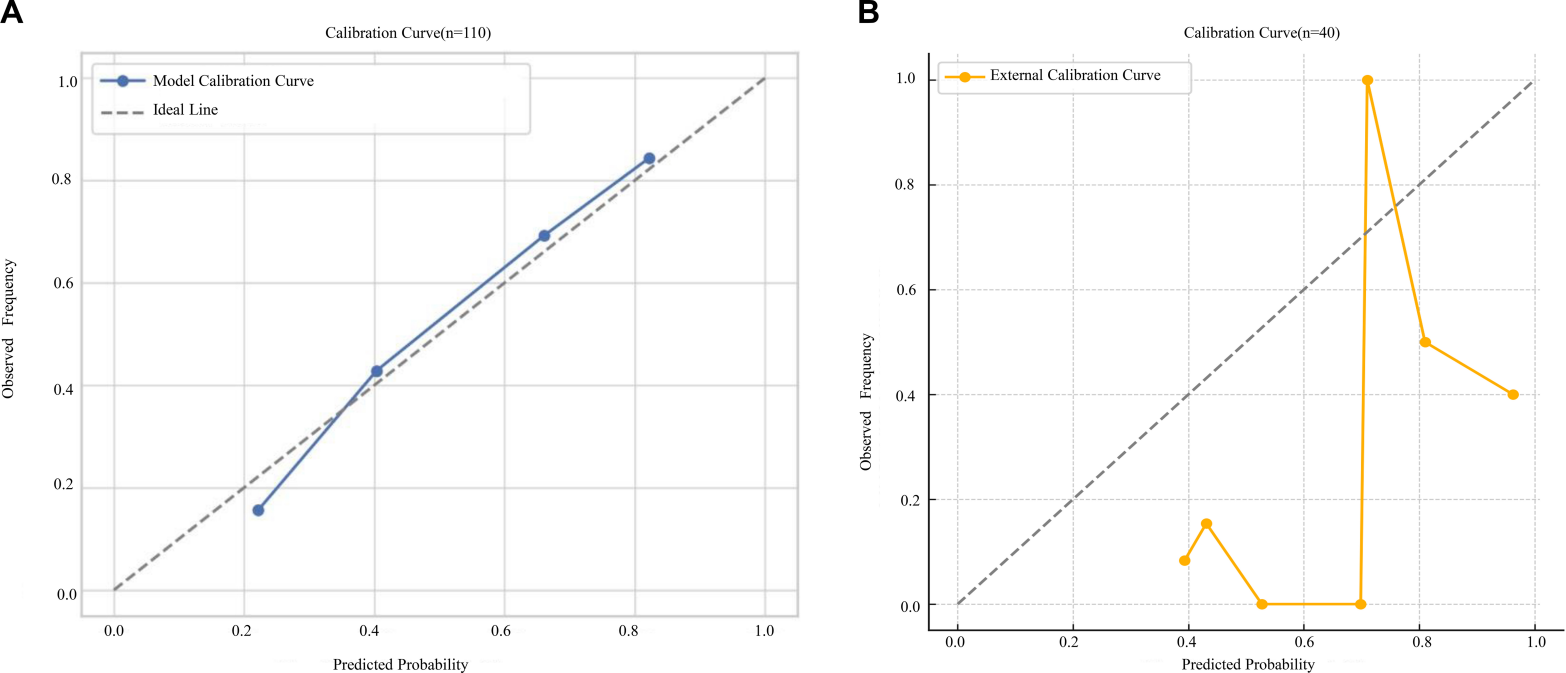
**(A)** Calibration curve of the predictive model in the Internal validation cohort. **(B)** Calibration curve of the prediction model in the external validation cohort.

The predictive model (CSF cell count + protein) was systematically evaluated using calibration curves and DCA. In the derivation cohort, the calibration curve demonstrated good agreement between predicted probabilities and observed outcomes, with the curve closely following the 45° reference line and only minor deviation at the lower end of the risk spectrum (**Figure 6A**). The apparent Brier score was 0.142, and after 1,000 bootstrap resamples the optimism-corrected Brier score was 0.148 (95% CI:0.139–0.158). The calibration intercept was −0.030 (95% CI: −0.210 to 0.150) and the calibration slope was 0.970 (95% CI: 0.860–1.090), indicating no evidence of systematic under- or over-prediction.

**Figure 7:**
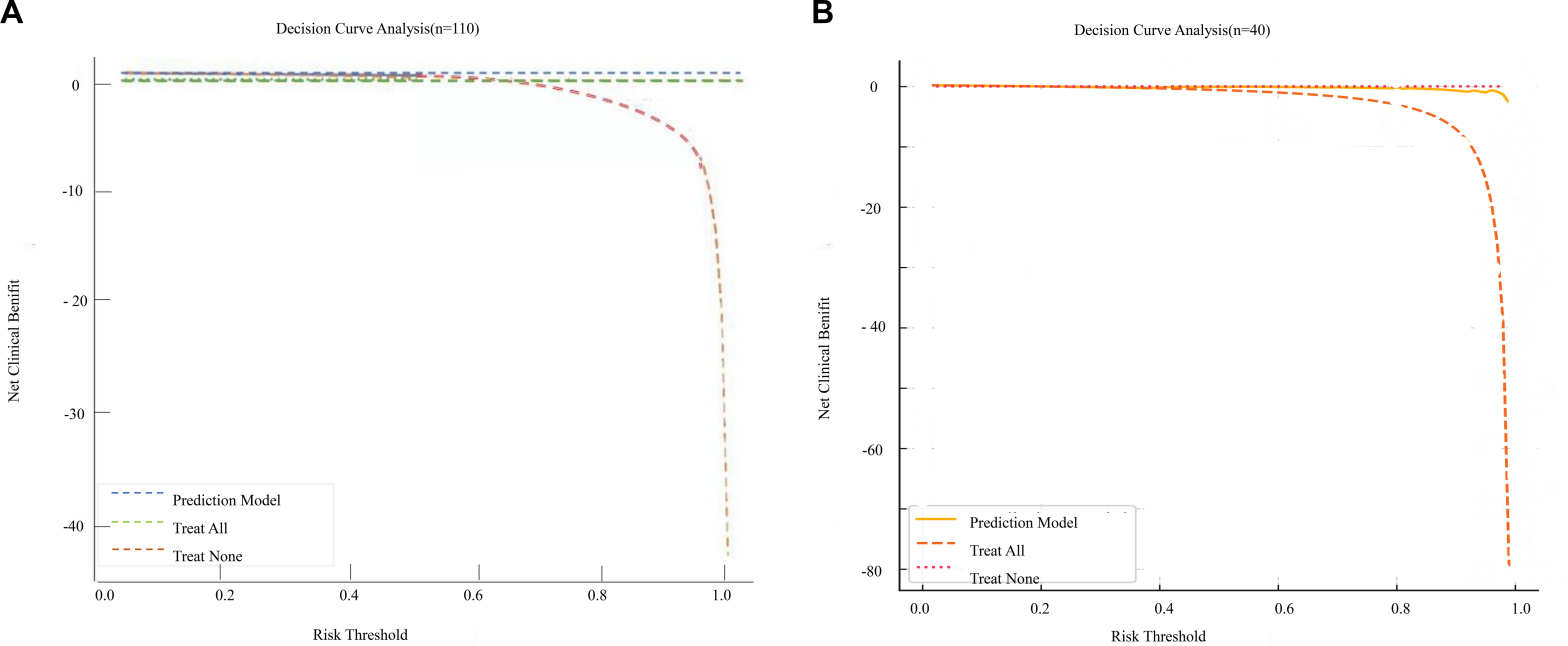
**(A)** DCA of the prediction model for clinical utility. **(B)** DCA of the prediction model in the external validation cohort.

DCA demonstrated that using the combined model to guide mNGS testing yielded a higher net benefit than the “test-all” or “test-none” strategies across a threshold probability range of 0.20–0.80 (**Figure 7A**), with a maximum net benefit of 0.28 at a threshold probability of 0.55 (95% CI 0.241–0.324). These findings support good calibration and meaningful clinical utility of the model in the derivation cohort (**Figure 7A**).

#### 6.5.2 External validation

A total of 40 patients were included in the external validation analysis. The calibration curve for the external validation cohort (**Figure 6B**) showed acceptable but more variable calibration, with slight deviations from the ideal line in some probability intervals, reflecting the limited sample size and wider uncertainty. In this cohort, the Brier score was 0.161 (95% CI:0.144–0.181), with a calibration intercept of 0.120 (95% CI: –0.340 to 0.590) and a calibration slope of 0.890 (95% CI:0.710–1.120), all derived from 1,000 bootstrap resamples. External validation with this independent cohort showed an AUC of 0.763 (95% CI:0.554–0.918), with a sensitivity of 77.8% and specificity of 67.7%. DCA in the validation cohort likewise confirmed clinical utility across threshold probabilities of 0.20–0.80 (**Figure 7B**), while the relatively wide confidence intervals highlight the need for cautious interpretation and further validation in larger cohorts.

### 6.6 Internal validation of the prediction model

**Figure 8:**
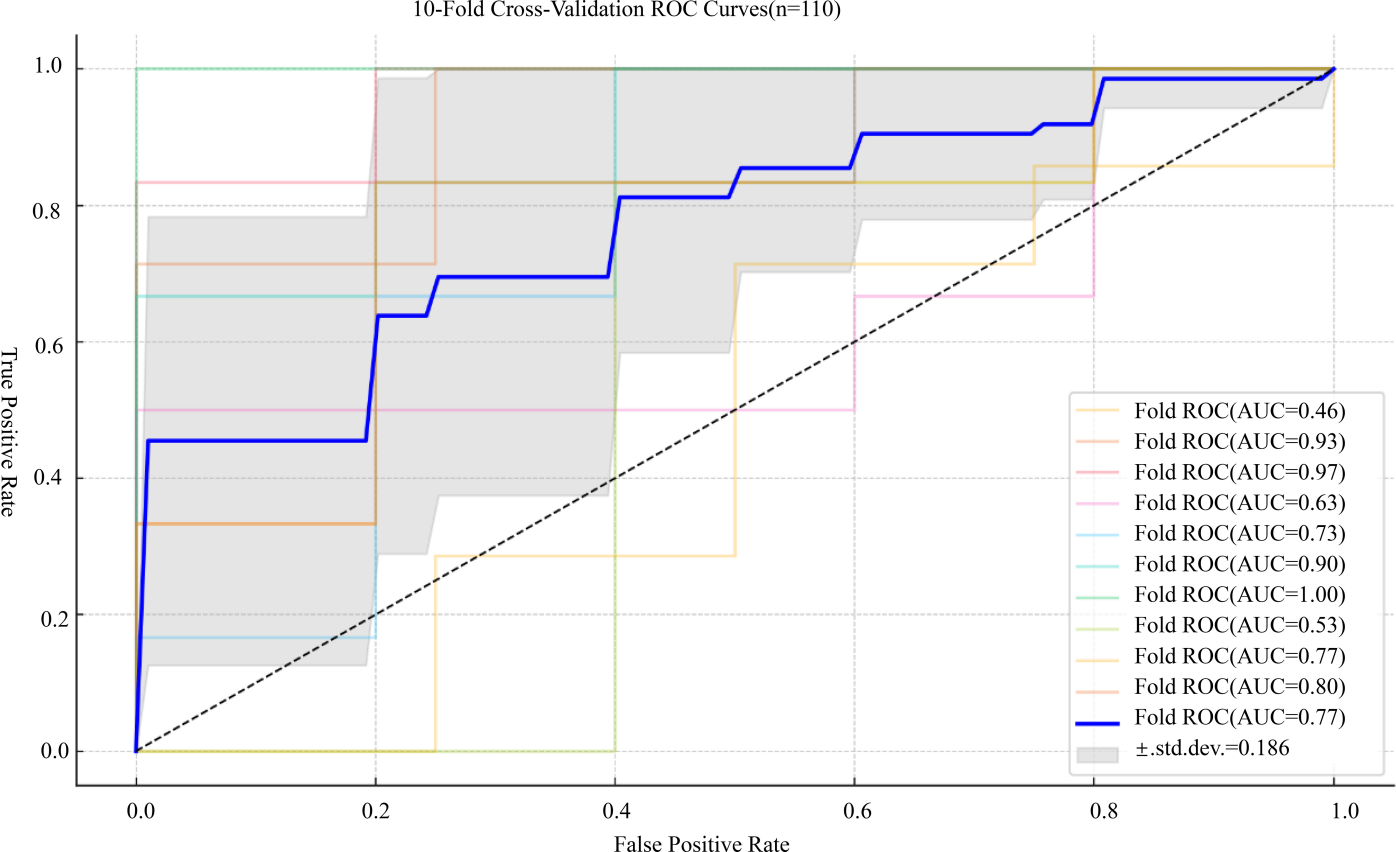
ROC curves generated from 10-fold cross-validation of the prediction model. The bold blue line represents the mean ROC curve from 10-fold cross-validation, with the grey shaded area indicating the standard deviation across folds; only the overall mean AUC with its 95% confidence interval is reported, rather than separate CIs for each fold.

**Figure 9:**
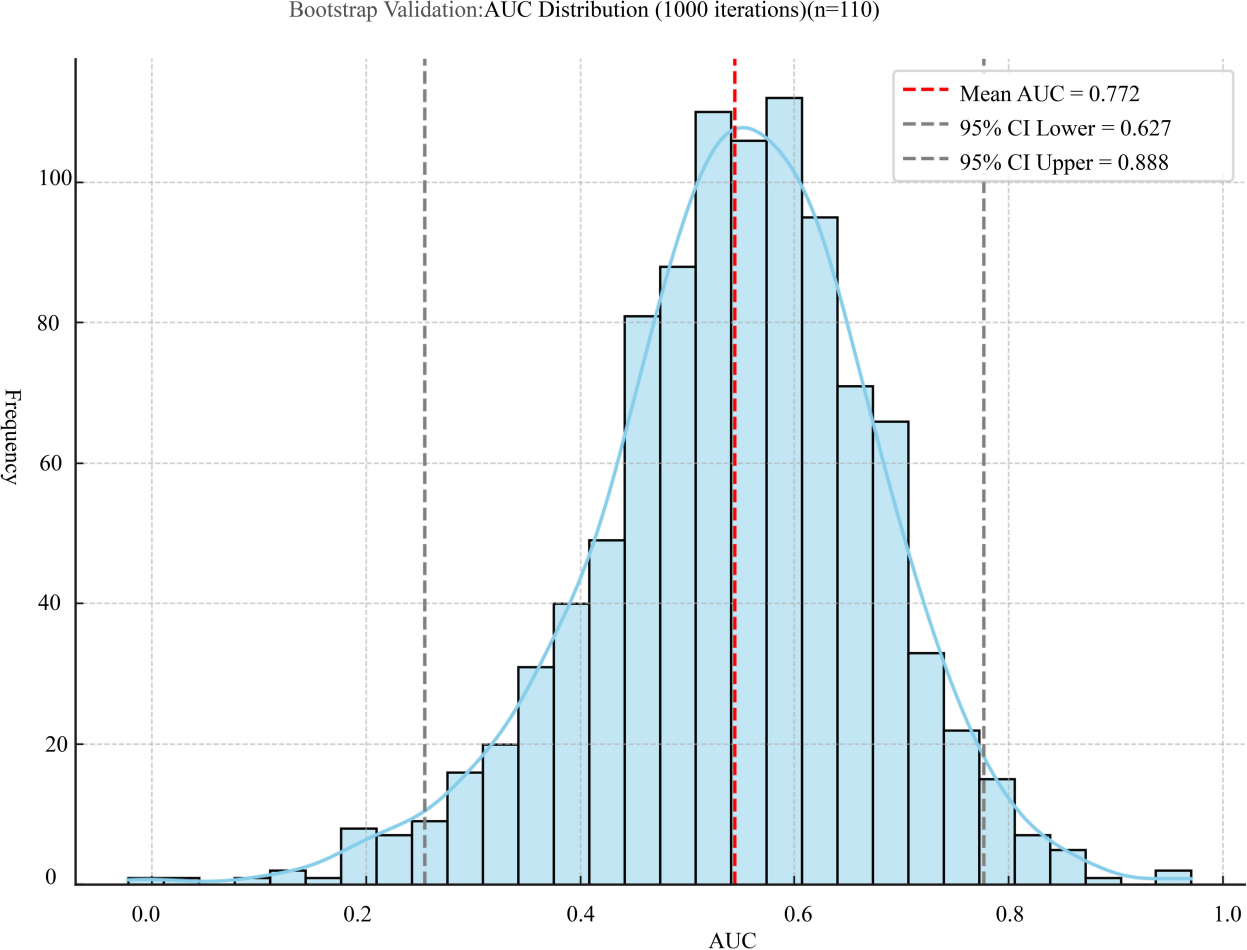
Distribution of AUC values from 1000 bootstrap iterations.

To further evaluate the robustness and generalizability of the prediction model, internal validation was conducted using 10-fold cross-validation and bootstrap resampling. The 10-fold cross-validation yielded a mean AUC of 0.773 ± 0.184, with a 95% CI of 0.641–0.904. In addition, the bootstrap method based on 1000 iterations produced a mean AUC of 0.770 ± 0.064, with a narrow 95% CI of 0.766–0.774, confirming the model’s consistent performance under repeated sampling (**Figures 8**, **9**).

### 6.7 Nomogram construction and clinical application

Based on the results of multivariate logistic regression analysis, a nomogram model was constructed to estimate the probability of mNGS positivity in CSF samples (**Figure 4**). The model incorporated two independent predictive factors: CSF cell count (cells/μL) and protein concentration (g/L). The nomogram assigns a point score to each variable. For example, a CSF cell count of 22 cells/μL corresponds to 60 points, and a protein concentration of 1.5 g/L corresponds to 40 points, yielding a total score of 100. This total score can then be used to estimate the probability of a positive mNGS result; a total score of 100 corresponds to an estimated probability of approximately 0.82. This nomogram provides a simple and intuitive tool for clinicians to perform individualized risk assessment before ordering mNGS testing. It may facilitate optimization of testing strategies, enhance diagnostic efficiency, and reduce unnecessary healthcare expenditure The nomogram was derived from a multivariable logistic regression model in which CSF cell count and protein concentration were entered as continuous predictors.

## 7 Discussion

With the expanding application of mNGS in infectious disease diagnostics, its value in CNS infections is increasingly recognized. Owing to its broad-spectrum detection capability, rapid turnaround time, and independence from predefined targets, mNGS has emerged as a powerful adjunct to conventional diagnostic methods (Schlaberg et al., 2017). However, its clinical use must be carefully balanced due to high costs, sensitivity to specimen quality, and potential for false positives (Batool and Galloway-Peña, 2023). In the present study, the mNGS positivity rate was 56.3%, markedly higher than that of conventional CSF culture (6.36%), with preliminary results available within 24 hours—significantly shorter than the 72–120 hours typically required for culture. These findings are consistent with previous studies (Gu et al., 2021; Tan et al., 2024), reinforcing the advantages of mNGS in detecting difficult-to-culture, mixed, or viral pathogens and supporting its broader adoption in CNS infection diagnostics.

Previous research has demonstrated a correlation between elevated CSF white blood cell counts, increased protein levels, and mNGS positivity (Wang et al., 2022; Yuan et al., 2024). Our results further support this association. mNGS-positive patients had significantly higher CSF cell counts, protein concentrations, and turbidity rates, along with lower glucose levels—suggestive of more intense inflammatory responses (Galea, 2021). These findings are consistent with known pathological mechanisms involving blood-brain barrier disruption, leukocyte infiltration, protein leakage, and glucose metabolism disturbance during CNS infections (Galea, 2021; Shahan et al., 2021). In addition, ICU admission rates, antimicrobial regimen adjustments, and mortality were all significantly higher in the mNGS-positive group, suggesting that mNGS positivity may reflect a higher likelihood of severe infection, warranting more aggressive clinical intervention.Logistic regression analysis identified abnormal CSF cell count and protein concentration as independent predictors of mNGS positivity (*P* < 0.05), underscoring the value of conventional CSF parameters in guiding mNGS testing decisions. Based on these findings, our predictive model and corresponding nomogram provide a practical, visual tool for individualized risk assessment. This may assist clinicians in optimizing mNGS testing strategies, improving diagnostic efficiency, and reducing unnecessary expenditures.

In current clinical practice, the decision to perform mNGS testing remains controversial. Challenges arise from national medical insurance reimbursement policies (Wang et al., 2025), high testing costs (Geng et al., 2021), inconsistent evaluations of clinical value (Chen et al., 2024), and the complex physician–patient relationship in China (Jing et al., 2017). Moreover, existing studies on the indications for mNGS testing have yielded inconsistent results (Graff et al., 2021; Deng et al., 2024).

In this study, comparative analysis of different variable formats revealed that models based on cell count and protein cut-offs, as well as combined models incorporating continuous and binary forms of these variables, achieved higher diagnostic performance. The AUC values for the cell cut-off, protein cut-off, cell + protein-CV, and cell + protein-BV models were 0.827, 0.813, 0.782, and 0.715, respectively, which were significantly higher than those of models based on cell-BV, protein-CV, and protein-BV (*P* < 0.05). These findings indicate that variable format has a substantial impact on model performance. Furthermore, combined models (CV/BV) demonstrated superior discrimination compared with single-indicator or purely binary models (*P* < 0.05), consistent with recent research suggesting that multiparameter models outperform traditional univariate approaches (Wang et al., 2021; Zhu et al., 2022).

Our CSF-based prediction model showed moderate discriminative performance and acceptable calibration. In the derivation cohort, the combined cell + protein (CV) model achieved an AUC of 0.782 with generally good apparent calibration; predicted probabilities broadly aligned with observed outcomes, with only slight deviation in the lower probability range. Internal validation using 10-fold cross-validation and bootstrap resampling yielded very similar average AUCs (0.773 ± 0.184 and 0.770 ± 0.064, respectively), indicating stable performance under repeated sampling. Internal validation using 10-fold cross-validation and bootstrap resampling yielded very similar average AUCs (0.773 ± 0.184 and 0.770 ± 0.064, respectively), supporting the stability of the model under repeated sampling.

To facilitate practical application, a visual nomogram was constructed based on the final logistic regression model. CSF cell count and protein concentration were assigned point values, allowing clinicians to estimate the risk of mNGS positivity—for example, a total score of 100 points corresponds to an approximately 82% probability. This tool supports individualised decision-making, especially in settings with limited access to mNGS or cost constraints, such as under China’s diagnosis-related groups (DRG)-based reimbursement system (Zhang and Sun, 2021). From a practical standpoint, risk-stratified use of the model may help refine mNGS ordering strategies: patients with markedly elevated CSF cell counts and protein levels who fall into a high-risk range (e.g. predicted probability ≥ 0.8) could be prioritised for immediate mNGS testing, particularly when conventional culture or PCR results are pending or inconclusive, whereas in patients with low predicted risk (e.g. probability < 0.2) and stable clinical status, clinicians may reasonably consider deferring or omitting mNGS testing to conserve resources without substantially compromising diagnostic yield.

Beyond the performance of the prediction model itself, it is important to recognise the intrinsic limitations of mNGS. Although mNGS offers high-throughput, broad-spectrum pathogen detection, challenges remain in quantitative interpretation, background contamination, and the lack of standardisation in bioinformatics pipelines and reporting thresholds (Greninger, 2018; Tsang et al., 2022). These issues must be considered when integrating model predictions and mNGS results into real-world clinical decision-making. In summary, both routine CSF analysis and mNGS play indispensable and complementary roles in the diagnostic workup of suspected CNS infections. By leveraging readily available CSF cell count and protein measurements, our prediction model and nomogram provide a simple, objective, and precision-oriented approach to stratify mNGS positivity risk and prioritise testing. This tool may contribute to more optimised diagnostic strategies, more efficient resource allocation, and improved clinical decision-making in CNS infections, while providing a theoretical basis and practical pathway for advanced diagnostic stewardship. Importantly, the model is intended to supplement rather than replace clinical judgement: in practice, the predicted risk should always be interpreted in the context of the overall clinical picture, routine CSF findings, imaging results, and guideline-based indications, and should not be used to deny mNGS in patients with strong clinical suspicion of CNS infection.

## 8 Limitation

This study has several limitations. First, it was conducted at a single tertiary hospital with a relatively small derivation cohort (n = 110), which may limit generalisability. External validation used a single-centre cohort of 40 patients that was treated as an independent test set and evaluated once without further resampling. The small external sample inevitably reduces statistical power, so the estimates remain imprecise and some instability cannot be excluded; larger, multicentre cohorts are therefore needed before the model can be adopted for routine clinical decision-making.

Second, as discussed above, the model provides decision support for prioritising mNGS testing but cannot replace clinician judgement or serve as a stand-alone rule.

Third, we only descriptively summarised the pathogen spectrum and typical CSF patterns and did not perform formal comparisons of CSF findings across specific pathogen classes (e.g. bacterial vs viral vs fungal), because subgroup sample sizes were small and any such analyses would have been seriously underpowered and at risk of incorporation bias. In addition, due to incomplete data, other potentially relevant clinical variables—such as C-reactive protein, procalcitonin, and detailed neuroimaging findings—could not be incorporated and may improve future models.

Finally, the “optimal” cut-offs for CSF cell count and protein (e.g. 21 × 10^6^/L and 1.5 g/L) were derived from ROC analysis by maximising the Youden index. These thresholds lie between adjacent observed values and should be regarded as sample-specific decision boundaries rather than universally applicable clinical thresholds. In **Figure 5**, ROC curves based on these dichotomised cut-offs show slightly higher apparent AUCs than the corresponding continuous predictors, which likely reflects optimism from selecting cut-offs on the same dataset and may not generalise to other populations. For this reason, the final multivariable model and nomogram are built on the underlying continuous variables, and cut-off-based AUCs should be interpreted as exploratory and illustrative only.

## Data Availability

Due to patient privacy and ethical restrictions, the raw sequencing data are not publicly available. De-identified clinical data and sequencing data can be made available from the corresponding authors upon reasonable request, subject to institutional/ethical approval.
